# Management of an Advanced Low‐Lying Implantation Disorder With Cervico‐Isthmic Features at 29 Weeks of Gestation Presenting With PPROM: A Case Report and Literature Review

**DOI:** 10.1155/crog/9537307

**Published:** 2026-09-20

**Authors:** Nafiseh Saedi, Azita Zare, Sara Ashtari, Nooshin Faraji, Fatemeh Golshahi, Behrokh Sahebdel, Elham Shirali, Sara Parviz, Mohammad M. Mehrabi

**Affiliations:** ^1^ Department of Obstetrics and Gynecology, Yas Hospital, Tehran University of Medical Sciences, Tehran, Iran, tums.ac.ir; ^2^ Breastfeeding Research Center, Family Health Research Institute, Imam Khomeini Hospital Complex, Tehran University of Medical Sciences, Tehran, Iran, tums.ac.ir; ^3^ Department of Radiology, Imam Khomeini Hospital, Tehran University of Medical Sciences, Tehran, Iran, tums.ac.ir

**Keywords:** cervico-isthmic pregnancy, cesarean scar pregnancy, endogenic cesarean scar pregnancy, low-lying ectopic pregnancy, low-lying implantation disorder

## Abstract

**Introduction:**

Cervico‐isthmic pregnancy (CIP) is a rare form of low‐lying implantation in which the gestational sac implants between the anatomical and histological internal os. It is associated with significant maternal morbidity due to abnormal placentation and a high risk of placenta accreta spectrum (PAS). Advanced cases beyond 20 weeks are exceedingly rare and pose major diagnostic and surgical challenges.

**Case Presentation:**

A 30‐year‐old woman, gravida 2, para 1, with a history of prior cesarean section and Asherman syndrome, presented at 28 weeks and 4 days of gestation with preterm premature rupture of membranes. An ultrasound revealed a viable breech fetus located in the lower uterine segment and cervico‐isthmic region. The presence of placental lacunae and bridging vessels suggested PAS. An MRI confirmed significant thinning of the lower uterine segment, nearly complete loss of myometrial tissue, and a preserved endocervical canal, consistent with CIP and PAS. Because of the high risk of hemorrhage, a cesarean section was performed using a posterior uterine incision, followed by immediate hysterectomy due to massive bleeding. Histopathological analysis confirmed the diagnosis of placenta increta in the cervico‐isthmic area. Both maternal recovery and neonatal outcomes were favorable.

**Discussion:**

This case highlights the diagnostic difficulty of advanced low‐lying implantation disorders with features favoring CIP. In late gestation, the anatomic boundaries among CIP, cervical pregnancy, and endogenic/Type I CSP may become difficult to define because the implantation site can expand beyond its original location. Careful evaluation of placental topography using ultrasound and MRI, supported by intraoperative anatomic findings and histopathologic correlation, can help characterize the implantation site and guide diagnostic interpretation. Nevertheless, even with multimodal assessment, overlap between CIP and endogenic/Type I CSP may persist, and definitive classification may remain challenging.

## 1. Introduction

Ectopic pregnancy refers to implantation outside the normal endometrial cavity. Cervico‐isthmic pregnancy (CIP) is a rare form of low‐lying implantation in which the gestational sac implants in the isthmic region, between the anatomical and histological internal os. CIP is considered a high‐risk and potentially life‐threatening pregnancy because the isthmic region has limited contractile capacity compared with the uterine corpus, impairing normal hemostasis after delivery or placental separation. In addition, CIP is frequently associated with abnormal placental adherence within the placenta accreta spectrum (PAS). Attempted placental removal in this setting may therefore result in massive hemorrhage [1, 2].

CIP has an incidence of 1:1800–1:2200 pregnancies, even though its true incidence is unknown because of the small number of cases reported in the literature [3]. The maximum gestational age reported in these pregnancies is 40 weeks. The management options depend on the gestational age at diagnosis. Most cases detected in the first trimester are misdiagnosed and treated as cervical pregnancies [4]. In the last 40 years, 19 cases of CIPs developing beyond 20 weeks have been described [1]. It is related to many complications, such as spontaneous miscarriage in the first or second trimester of pregnancy, preterm labor, uterine rupture, massive hemorrhage, hysterectomy, and maternal mortality [3].

## 2. Case Presentation

A 30‐year‐old gravida 2, living 1, previous cesarean Section 1 woman at 28 weeks and 4 days of gestation presented to the emergency department at Yas Hospital, Tehran, Iran, with preterm premature rupture of membranes. The patient had undergone a cesarean Section 5 years earlier without complications. Following delivery, she reported markedly reduced menstrual flow, mainly characterized by occasional spotting rather than normal cyclic bleeding. Three years after delivery, she was diagnosed with Asherman syndrome based on diagnostic evaluation and hysteroscopy. Despite hysteroscopic and medical management, minimal menstrual bleeding persisted. Conception subsequently occurred spontaneously.

During the current pregnancy, noninvasive prenatal testing was reported to be low risk, with a fetal fraction of 10%. First‐ and second‐trimester ultrasound examinations were performed at outside centers. At 7 weeks of gestation, low placentation of the gestational sac was reported, and the residual myometrial thickness at the prior cesarean scar was measured at 5 mm. However, at 12 weeks of gestation, the placenta was described as fundal, and the nuchal translucency was 1.3 mm. At 18 weeks of gestation, an anomaly scan revealed two 5 cm subchorionic hematomas, without associated vaginal bleeding. The available external reports did not describe sonographic features suggestive of PAS.

The patient was first evaluated at our tertiary center at 28 weeks and 4 days of gestation. She reported fluid leakage for approximately 3 weeks before admission. On physical examination, her heart rate was 110 beats per minute, blood pressure was 125/75 mmHg, oral temperature was 37.4°C, and respiratory rate was 17 breaths per minute. Speculum examination revealed pooling of amniotic fluid, confirming rupture of membranes. The cervix was closed, and no vaginal bleeding was noted. The initial nonstress test was reactive, without uterine contractions.

Ultrasound examination at our center demonstrated a single breech fetus with a right anterolateral placenta. The estimated fetal weight was 1260 g, corresponding to the 45th percentile. The amniotic fluid index was 5 cm, and the biophysical profile score was 10/10. Initial laboratory investigations revealed hemoglobin 8.9 g/dL, white blood cell count 13,000/*μ*L, platelet count 181,000/*μ*L, prothrombin time 13 s, partial thromboplastin time 25 s, INR 1.0, C‐reactive protein 8 mg/L, erythrocyte sedimentation rate 24 mm/h, serum iron 179 *μ*g/dL, total iron‐binding capacity 419 *μ*g/dL, and ferritin 19 ng/mL.

The patient underwent expectant management for preterm premature rupture of membranes. She received two doses of 12 mg betamethasone for fetal lung maturation, magnesium sulfate for fetal neuroprotection, and prophylactic antibiotics with ampicillin and azithromycin.

**Figure 1:**
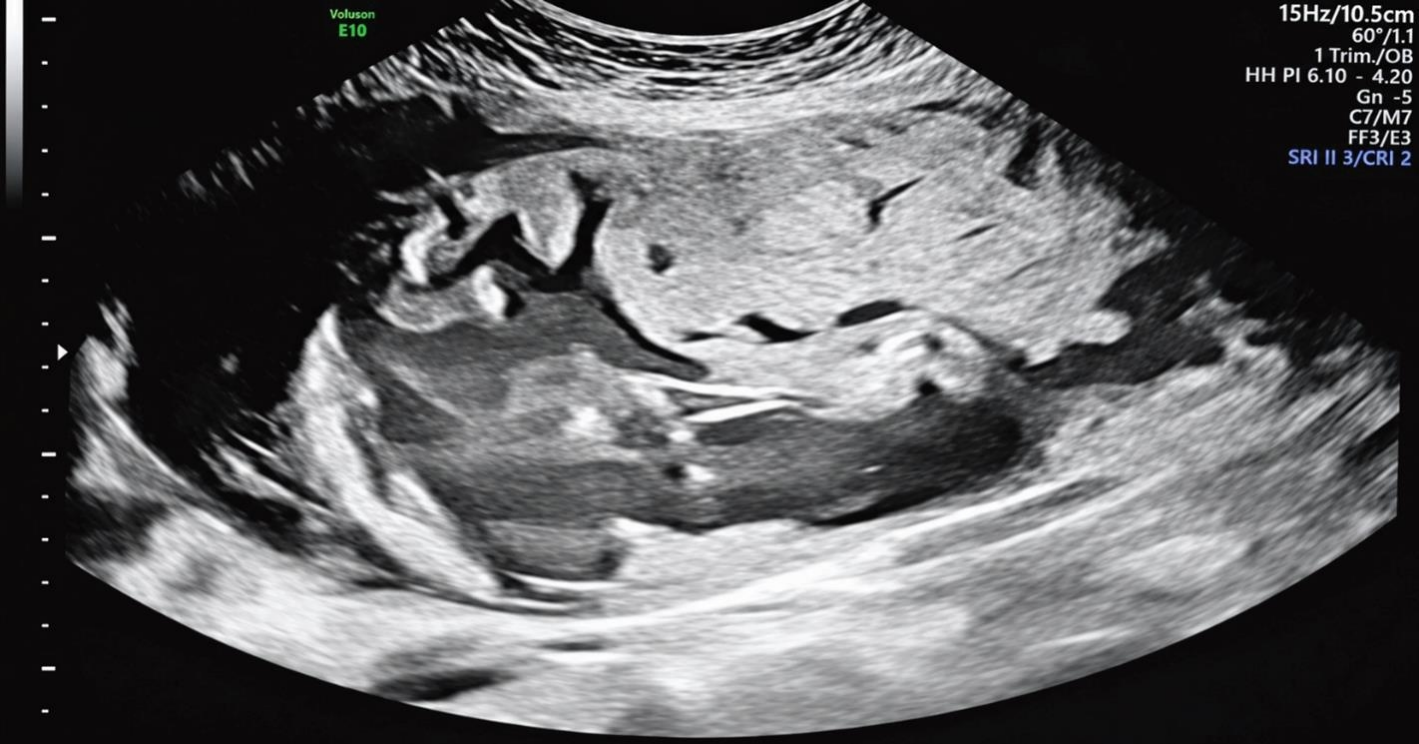
Ultrasound features suspicious for placenta accreta spectrum. Gray‐scale ultrasound demonstrates an abnormally thickened low‐lying anterior placenta with multiple placental lacunae and an intraplacental hematoma.

A repeat detailed ultrasound at our center demonstrated an abnormally located anterior placenta with multiple placental lacunae, intraplacental hematomas, and prominent bridging vessels extending toward the uterovesical interface, raising strong suspicion for PAS, particularly placenta increta (Figure 1). She received three units of packed red blood cells, which increased her hemoglobin level to 9.4 g/dL.

**Figure 2:**
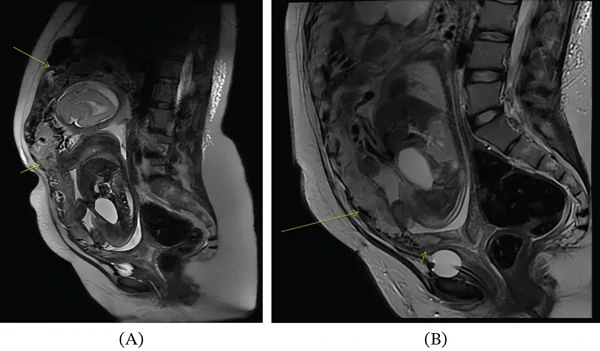
MRI features of low‐lying placentation and suspected placenta accreta spectrum. (A) Sagittal T2‐weighted MR image showing the predominantly anterior placenta with marked thinning and near‐complete loss of the overlying lower uterine‐segment myometrium (short arrow), with a relatively preserved fundal myometrial mantle (long arrow). (B) Sagittal T2‐weighted MR image demonstrating prominent vessels along the anterior lower uterine segment (long arrow) and marked involvement of the anterior cervico‐isthmic region (short arrow).

A 1.5‐Tesla MRI without contrast was performed on a GE scanner to evaluate the abnormal implantation site further and assess potential complications related to placental invasion. Sagittal T2‐weighted images demonstrated a viable intrauterine fetus (Figure 2). The placenta was predominantly anterior and appeared abnormally adherent to the lower uterine segment. The myometrium overlying the lower uterine segment and cervico‐isthmic region was markedly thinned, with focal loss of the normal hypointense myometrial layer and areas of near‐complete myometrial loss, raising concern for PAS, particularly placenta increta.

**Figure 3:**
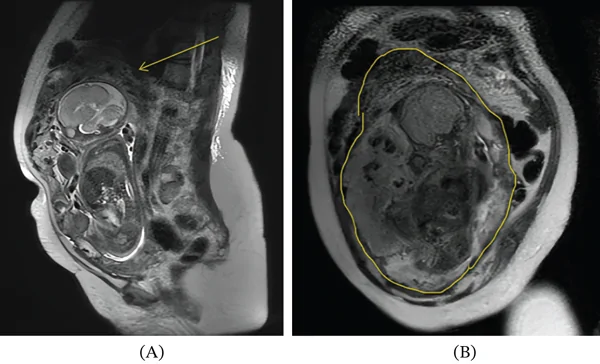
MRI demonstration of the low‐lying gestational configuration. (A) Sagittal T2‐weighted MR image showing a prominent fundal myometrial mantle with no well‐defined gestational sac in the expected fundal uterine cavity (arrow). (B) Coronal T2‐weighted MR image delineating the pear‐shaped uterine configuration and the low‐lying position of the gestational sac.

The bulk of the gestational sac and fetus was positioned within the lower uterine segment and isthmic region, extending toward the anterior uterine wall. One of the typical features of CIP is an empty uterine fundus. However, at this advanced gestational age, distinguishing between a truly empty fundus and fundal compression by an enlarging pregnancy can be challenging. In this patient, the fundal myometrium appeared thinned and displaced superiorly, forming a pear‐shaped uterine configuration, and no well‐defined gestational sac was identified in the expected fundal position (Figure 3).

**Figure 4:**
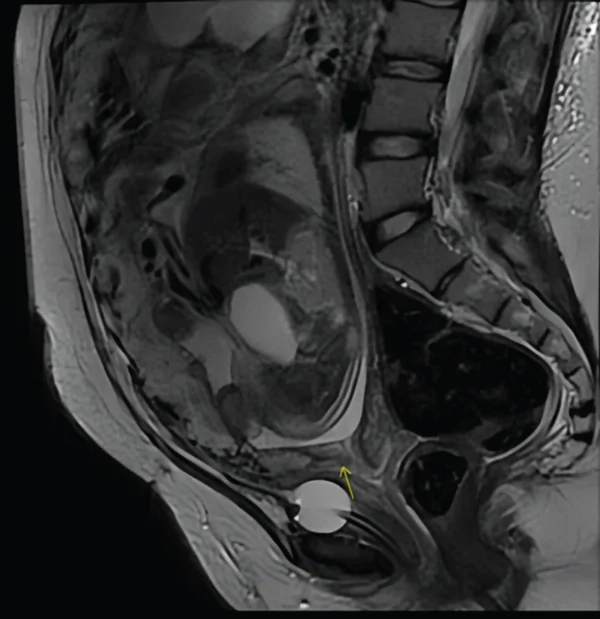
MRI appearance of the cervico‐isthmic region. Sagittal T2‐weighted MR image demonstrates cervical shortening and effacement with preservation of an identifiable endocervical canal free of gestational tissue.

The cervix was significantly shortened and effaced, suggesting advanced cervical remodeling. However, the endocervical canal remained identifiable and free of a gestational sac, making primary cervical pregnancy unlikely (Figure 4). No significant free fluid, extrauterine hematoma, or uterine rupture was noted at the time of imaging. Nevertheless, the combination of low‐lying implantation, a thinned lower uterine segment, and suspected abnormal placentation placed the patient at high risk for massive hemorrhage and uterine rupture.

**Figure 5:**
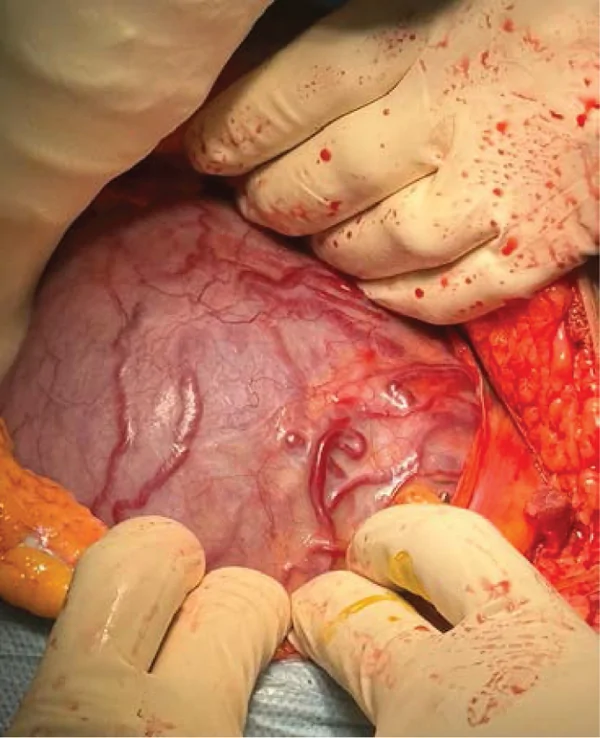
Intraoperative hypervascularity of the lower uterine segment. Intraoperative appearance demonstrates marked surface vascularity over the anterior lower uterine segment and low‐lying implantation region.

**Figure 6:**
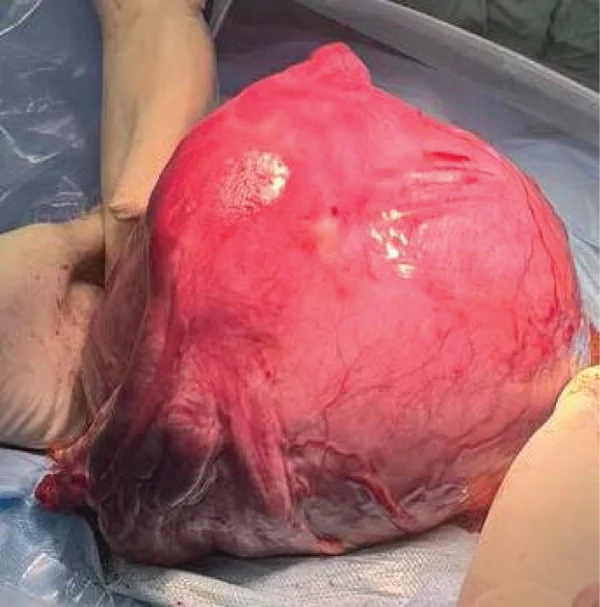
Intraoperative configuration of the low‐lying implantation. Following uterine exteriorization, marked distension of the lower uterine segment and cervico‐isthmic region is seen with a comparatively unexpanded fundus, supporting a predominantly cervico‐isthmic implantation pattern.

Given the high risk of catastrophic hemorrhage, surgical delivery was planned. Under general anesthesia and through a midline skin incision, the anatomical layers of the abdominal wall were opened in order. Marked uterine vascularity was evident intraoperatively (Figure 5). The uterus was exteriorized from the abdomen. Intraoperatively, the uterine fundus appeared empty, and placental implantation was predominantly located in the isthmic region, superior to the anatomical internal os, extending into the cervico‐isthmic area rather than being confined to a discrete cesarean scar niche (Figure 6). Severe adhesions between the bladder and the anterior uterine wall were noted.

Because of the extensive anterior placental involvement and prominent vascularity, a posterior uterine incision was performed to avoid transplacental entry and reduce the risk of catastrophic hemorrhage. A breech female neonate weighing 1470 g was delivered, with Apgar scores of 5 and 8 at one and 5 min, respectively. The neonate was intubated immediately for respiratory support.

**Figure 7:**
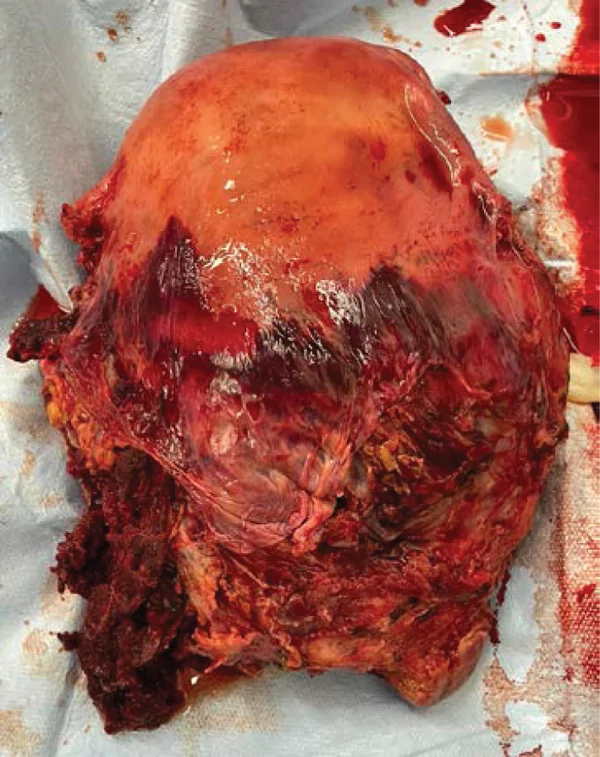
Gross hysterectomy specimen following cesarean delivery. The specimen demonstrates marked hemorrhagic involvement and distortion of the lower uterine segment and cervico‐isthmic region.

Due to significant intraoperative hemorrhage, an immediate total hysterectomy was performed (Figure 7). Intraoperatively, the patient received five units of packed red blood cells, five units of fresh frozen plasma, five units of platelets, and 2 g of tranexamic acid. A pelvic drain was placed at the end of surgery.

After surgery, the patient was transferred to the intensive care unit. Postoperatively, she stabilized without further complications. Two days later, she was transferred to the general ward. Inflammatory marker levels normalized. She remained afebrile and hemodynamically stable, with a heart rate of 78 beats per minute, a blood pressure of 120/70 mmHg, a temperature of 37.0°C, and a respiratory rate of 17 breaths per minute. Repeat laboratory tests showed hemoglobin 9.2 g/dL, white blood cell count 14,400/*μ*L, and platelet count 283,000/*μ*L. The pelvic drain was removed on postoperative Day 3. The patient was discharged in stable condition 5 days after surgery.

Histopathologic examination revealed that the placenta was predominantly located in the cervico‐isthmic region, involving the lower uterine segment and extending into the upper endocervical canal. Placental tissue was firmly adherent to the underlying myometrium at the site corresponding to the previous cesarean section scar. The lower uterine segment was involved up to the serosal surface. Extensive hemorrhage and clotted blood were present.

Microscopically, sections from the lower uterine segment and cervico‐isthmic region demonstrated chorionic villi directly invading the myometrium, with no intervening decidua. The villi infiltrated fibrotic scar tissue consistent with the prior cesarean section. Marked vascular congestion, extensive hemorrhage, and focal necrosis were observed. In the cervico‐isthmic region, the myometrium at the implantation site was markedly thinned or absent. Sections obtained from the upper uterine body showed normal endometrium and myometrium without evidence of placental attachment. These findings were consistent with PAS, placenta increta, corresponding to grade 3A involvement. During follow‐up, the neonate was discharged after a 2‐month stay in the neonatal intensive care unit.

## 3. Discussion

This case illustrates the clinical challenges of an advanced low‐lying implantation disorder complicated by PAS, PPROM, severe hemorrhage, and cesarean hysterectomy. The overall imaging, intraoperative, and histopathologic findings suggested predominant involvement of the lower uterine segment and cervico‐isthmic region, with preservation of the endocervical canal and no placental attachment in the upper uterine body. These features supported CIP as the favored topographic diagnosis over true cervical pregnancy, while remaining within the recognized continuum of low‐lying implantation disorders. Given the prior cesarean delivery and histologic involvement of fibrotic scar tissue, some diagnostic overlap with advanced endogenic/Type I cesarean scar pregnancy (CSP) remains possible.

CIP occupies a complex position within the broader spectrum of low‐lying implantation disorders. Although CIP is not separately categorized in the European Society of Human Reproduction and Embryology (ESHRE) terminology, which consolidates uterine ectopic pregnancies into cervical pregnancy and CSP, this simplified classification does not necessarily exclude the concept of a cervico‐isthmic implantation site [5, 6].

Tsai et al. introduced the concept of “low‐lying implantation ectopic pregnancy” (LLIEP) to encompass CSP, cervical pregnancy, and CIP within a unified spectrum of abnormal implantation in the lower uterine segment. Within this framework, the defining criterion is not historical classification but precise anatomic localization relative to the internal os, cervical canal, and uterine cavity [6]. It is also emphasized that CIP is characterized by implantation in the isthmic region superior to the anatomical internal os, with a closed and preserved cervical canal and limited or absent involvement of the upper uterine cavity [7].

In the present case, the preserved cervical canal, absence of placental attachment in the uterine fundus, and implantation centered in the cervico‐isthmic zone support classification as CIP rather than primary CSP or true cervical pregnancy, while acknowledging potential overlap with endogenic CSP in advanced gestation [8]. Although histopathology demonstrated villous invasion into fibrotic tissue corresponding to a prior cesarean scar, scar involvement alone does not mandate a diagnosis of CSP.

CSP is reported more frequently than CIP [9]. Vial et al. described two types of CSP, distinguishing endogenic forms that grow toward the uterine cavity from exogenic forms that progress toward the bladder. However, both originate within a discrete scar niche. In contrast, our findings demonstrated a broad implantation bed spanning the cervico‐isthmic region rather than a gestational sac embedded primarily within a defined scar defect [10]. Tolino et al. acknowledged that diagnostic overlap between CIP and CSP may occur, particularly in advanced gestations, reinforcing the notion of a continuum rather than strictly separable entities. However, when implantation predominance lies between the histological and anatomical internal os and the cervical canal remains preserved, the topographic criteria favor CIP [3].

The subsequent clinical course was also consistent with published observations of advanced CIP. ISUOG guidance highlights that CIP may progress beyond the first trimester and is associated with a substantial risk of PAS and severe hemorrhage [7]. In this patient, the development of placenta increta is pathophysiologically consistent with current models of PAS, in which abnormal trophoblastic invasion is attributed to disruption of the endometrial–myometrial interface, particularly in areas of prior uterine surgery [11, 12]. Both previous cesarean section and intrauterine adhesions may impair decidualization and create focal areas of deficient decidua basalis, thereby facilitating direct villous invasion into the myometrium. Although CSP is also strongly associated with PAS, the risk profile alone cannot determine the diagnosis. Accurate interpretation, therefore, requires detailed topographic assessment, as emphasized by the ESHRE Working Group [5, 13].

The imaging trajectory in this case underscores the diagnostic challenges of low implantation in early gestation. Initial examinations outside the tertiary center noted low placentation and reduced myometrial thickness at the prior cesarean site, but did not raise a strong suspicion for invasive placentation. As gestation progressed, targeted ultrasound revealed multiple placental lacunae, subplacental hypervascularity, and bridging vessels toward the bladder, features recognized as highly suggestive of PAS [14]. MRI subsequently demonstrated marked thinning of the lower uterine segment and near‐complete loss of the myometrial–placental interface, further supporting invasive placentation and clarifying the spatial relationship between the placenta, isthmus, cervix, and prior scar. MRI is widely accepted as a complementary modality when ultrasound findings are equivocal or when surgical planning requires detailed topographic mapping [15, 16]. In this case, MRI also helped argue against a true cervical pregnancy by showing that the gestational sac did not occupy the endocervical canal.

A review of previously reported cervico‐isthmic pregnancies progressing beyond 20 weeks of gestation, summarized in Table 1, provides important comparative context. Since early descriptions in the 1980s, approximately 20 advanced or viable cases have been documented. Gestational age at delivery has ranged from the late second trimester to term, and live birth has been achieved in the majority of cases. Neonatal outcome appears to be primarily determined by gestational age at delivery rather than by the implantation site itself [19, 20, 25, 30]. A consistent theme across these reports is diagnostic uncertainty in early pregnancy, with several cases initially misclassified as cervical pregnancy or CSP before redefinition intraoperatively or histologically. This pattern supports the LLIEP spectrum concept proposed by Tsai et al. and highlights the necessity of systematic landmark‐based assessment [6].

**Table 1:** Published cases of cervico‐isthmic pregnancy progressing beyond 20 weeks of gestation: clinical characteristics, management, and outcomes.

	Authors	Year	Age (year)	G.A (week)	Gravidity	CC	Shortened CL	Treatment	Fetal outcome	Blood transfusion	Diagnosis confirm
1	David et al. [17]	1980	NA	40 weeks	NA	LP		CS, hysterectomy	3700 g, alive	—	During operation
						VB					
2	Cohen et al. [18]	1985	36	27 weeks	PG	Painless VB	+cerclage	CS, hysterectomy	Male	5 U P.C	During operation
						Then, LP, ROM			1050 g, alive		
3	Jelsema and Zuidema [19]	1992	30	38 weeks	Multiparous	NA	+ [20]	CS, hysterectomy	3100 g, alive	Blood transfusion [19]	5.5 weeks: CP Pathology report: CIP
4	Iloabachie et al. [21]	1993	NA	37 weeks	PG	VB	+ [20]	CS	Female 2500 g, alive male 2200 g, alive	Blood transfusion [19]	During operation [19]
				Twin							
5	Wehbé [22]	1993	NA	24 weeks	G3 p3 cs1	NA		CS hysterectomy	NA	NA	First trimester
6	Nicola Strobelt [1]	1999	41	30 weeks	G6 P2 Ab3 D&C3	VB, LP	+	Cs, hysterectomy	Female 1450 g alive bilateral clubfoot	7 U P.C 10 U FFP	6.5 weeks
7	Spryros A. Mesogitis [23]	2001	26	37 weeks + 2 days	PG	PPROM, LP		NVD with vacuum, delivered placenta, hysterectomy	Male 2750 g	4 U whole blood 8 U PLT	During operation
8	Itakura [24]	2003	33	23 weeks [19]	NA	VB [19]		CS MTX, UAE	NA	Blood transfusion [19]	21weeks (MRI)
9	Tetsuro Honda [25]	2005	39	32 weeks	PG, IVF	NA	+ [20]	CS, hysterectomy	Alive neonate	Autologous blood transfusion	6 weeks
10	Gilles Kayem [26]	2008	32	34 weeks	G2 L1 CS1	PPROM (25 weeks), VB		CS	Female, 2340 g, alive	—	During operation
11	Avery, Daniel M. [27]	2009	35	38 weeks + 3 days	G3 P2 CS2	VB		CS, hysterectomy	NA	20 U P.C	5 weeks + 6 days: CP pathology report: CIP
										8 U FFP	
										CRYO	
										Activated factor IIV	
12	Atsuhiko Sakai [19]	2012	29	37 weeks	NA	NA		CS MTX, UAE	NA	—	8 weeks
13	Alexander Weichert [28]	2012	35	34 weeks	G3 P1 Ab2 D&C	Painless VB	+	CS, hysterectomy	Female 1645 g	6 U P.C	19 weeks + 5 days sonography and MRI
										8 U FFP	
14	Hiroko Yoshida [20]	2014	39	37 weeks	G3 p2 Ab1 D&C	Elective CS	+cerclage	CS, hysterectomy	Female 2600 g	1600 mL of autologous blood	8 weeks
15	Chisa Ito [29]	2023	33	34 weeks + 1 days	Multiparous CS1	Elective CS CSP	+	CS, hysterectomy	Male infant 1938 g	6 U P.C	7 weeks: CSP during operation: CIP
16	Jinkyeong Ha, MD [30]	2023	35	31 weeks + 4 days	G3 P3 L1 CS2	VB		CS, hysterectomy	1920 g, alive	11 U P.C	8 weeks + 4 days
										9 U FFP	
						LP					
17	Jinkyeong Ha, MD [30]	2023	35	35 weeks	G5 P3 Ab2 L2 CS2	VB		CS, hysterectomy	2730 g alive	9 U P.C	7 weeks
										6 U FFP	
						LP					
										7 U PLT	
18	Jinkyeong Ha, MD [30]	2023	37	33 weeks	G3 Ab2	VB		CS	2150 r, alive	4 U P.C	5 weeks + 6 days
						PPROM				3 U FFP	
19	Jinkyeong Ha, MD [30]	2023	36	38 weeks + 6 days	G4 P2 Ab1 L0 CS2	Elective operation		CS	4140 g alive	5 U P.C	6 weeks + 2 days
20	This case	2025	30	29 weeks	G2 L1 CS1	PPROM		CS, hysterectomy	Female, 1470 g	8 U P.C	During operation
										5 U FFP	
						VB				5 U PLT	

Abbreviations: Ab, abortions; CC, chief complaint; CIP, cervico‐isthmic pregnancy; CL, cervical length; CS, cesarean section; CSP, cesarean scar pregnancy; D&C, dilatation & curettage; FFP, fresh frozen plasma; IVF, in vitro fertilization; L, living children; LP, labor pain; MTX, methotrexate; NA, not available; NVD, normal vaginal deliver; P.C, packed cells (blood transfusion); PG, pregnancy (parity not fully defined in source); PLT, platelets (blood transfusion); ROM, rupture of membranes; UAE, uterine artery embolization; VB, vaginal bleeding.

Another recurrent finding in advanced CIP is the high incidence of abnormal placentation and severe hemorrhage. Multiple case reports document massive transfusion requirements and frequent need for cesarean hysterectomy [25, 28, 30]. Although advanced CIP can be compatible with fetal viability, it is rarely benign from a maternal standpoint. The coexistence of PAS appears to be a central determinant of hemorrhagic severity.

Preterm complications in CIP are heterogeneous. Some pregnancies are complicated by recurrent vaginal bleeding, cervical shortening, or the need for cerclage, whereas others may progress without major symptoms until late gestation. In the present case, prolonged PPROM at 28–29 weeks culminated in delivery at 29 weeks, with MRI evidence of cervical shortening and effacement. Strobelt et al. and Weichert et al. suggested that implantation in the cervico‐isthmic region may predispose to bleeding and preterm birth because of the structural vulnerability of the lower uterine segment and its proximity to the internal os [1, 28]. Cohen et al. and Ha et al. similarly reported diverse pathways to prematurity, including bleeding and membrane rupture [18, 30]. Together, these reports suggest that preterm delivery in CIP may result from multiple mechanisms, including bleeding, membrane rupture, and cervical remodeling. In contrast, hemorrhagic severity appears to be driven primarily by invasive placentation and lower uterine segment involvement rather than cervical length alone.

Established PAS principles guided surgical management in this case. Transplacental uterine incision and attempted placental separation are recognized precipitants of catastrophic hemorrhage. Preoperative imaging revealed extensive anterior placental involvement with marked hypervascularity, and intraoperative inspection confirmed dense adhesions and prominent vascular structures in the anterior lower uterine segment. A posterior uterine incision was therefore selected to avoid traversing the placental bed and to permit fetal extraction remote from the area of invasion. This strategy reflects individualized operative planning based on placental topography, depth of invasion, and surgical exposure, rather than a standardized technique. Despite careful planning, a massive hemorrhage necessitated immediate total hysterectomy, consistent with published experiences in advanced CIP complicated by PAS [25].

The patient’s fertility history adds further pathophysiologic insight. She had a history consistent with Asherman syndrome following cesarean delivery, with persistent hypomenorrhea despite treatment, followed by spontaneous conception. Asherman syndrome is characterized by intrauterine adhesions that impair endometrial regeneration and receptivity, often resulting in infertility or adverse reproductive outcomes [31]. Pregnancy rates after adhesiolysis vary depending on adhesion severity, and recurrence is common. Siferih et al. reported lower conception rates in low‐resource settings, particularly among those with severe disease [32]. Disruption of the endometrial–myometrial interface in Asherman syndrome may create areas of deficient decidualization, analogous to cesarean scar defects, thereby predisposing to abnormal implantation and invasive placentation [11]. The convergence of prior cesarean scar, intrauterine adhesions, low implantation, and PAS in this patient supports the hypothesis that postsurgical uterine pathology may shift implantation toward the lower uterine segment and increase the risk of LLIEP and PAS.

## 4. Conclusion

This case highlights the significant diagnostic and surgical challenges posed by advanced low‐lying implantation disorders complicated by PAS and PPROM. Although the overall clinical, imaging, intraoperative, and histopathologic findings favored a cervico‐isthmic pattern of implantation, overlap with advanced endogenic/Type I CSP could not be completely excluded, underscoring the continuum that exists among low‐lying implantation disorders. Comprehensive assessment of implantation topography using ultrasound and MRI is critical for anticipating PAS, guiding surgical planning, and reducing the risk of catastrophic hemorrhage. This case further demonstrates that advanced low‐lying implantation disorders are associated with substantial maternal morbidity and frequently require complex multidisciplinary management, including cesarean hysterectomy. Early recognition, referral to specialized tertiary centers, and meticulous preoperative preparation remain essential to optimize maternal and neonatal outcomes.

**Nomenclature**
AFI: amniotic fluid index
ART: assisted reproductive technology
BP: blood pressure
bpm: beats per minute
BPP: biophysical profile
CC: chief complaint
CIP: cervico‐isthmic pregnancy
CP: cervical pregnancy
cryo: cryoprecipitate
CS: cesarean section
CSP: cesarean scar pregnancy
D: days
FFP: fresh frozen plasma
G: gravida
GA: gestational age
gr: gram
Hb: hemoglobin
HR: heart rate
ICU: intensive care unit
IVF: in vitro fertilization
L: live
LLIEP: low‐lying implantation ectopic pregnancy
LP: labor pain
mm: millimeter, cm: centimeter
MRI: magnetic resonance imaging
NICU: neonatal intensive care unit
NIPT: noninvasive prenatal testing
NST: nonstress test
NT: nuchal translucency
P: para
P.C: packed red blood cells
plt: platelets
PPROM: premature rupture of membranes
ROM: rupture of membrane
RR: respiratory rate
T: temperature
U: unites
UAE: uterine artery embolization
VB: vaginal bleeding
W: weeks
WBC: white blood cell

## Author Contributions

N.S., A.Z., and N.F. contributed to the study idea, supervision, data collection, and case management. N.F., E.S., and M.M.M. contributed to manuscript writing, visualization, and editing. A.Z. and B.S. contributed to data collection, case management, and processing. N.S., S.A., and F.G. contributed to original draft preparation, methodology, resources, and manuscript review.

## Funding

No funding was received for this manuscript.

## Disclosure

All authors read, edited, and approved the final manuscript. Sara Parviz had full access to all data in this study and takes full responsibility for the data′s integrity and the accuracy of the analysis. Sara Parviz affirms that this manuscript is an honest, accurate, and transparent account of the case being reported; that no important aspects of the case have been omitted; and that any discrepancies from the case as planned, and if relevant, registered, have been explained. As this is a single case report, no formal statistical analysis was performed. No statistical tests, subgroup analyses, or exploratory statistical analyses were conducted, and no statistical software was used.

## Ethics Statement

This study was approved by the ethics committee affiliated with Tehran University of Medical Sciences. This case report was prepared in accordance with the CARE reporting guidelines.

## Consent

The patient provided written informed consent for participation and publication of this case report and associated clinical images. The study procedures were conducted in accordance with the Declaration of Helsinki and applicable institutional and national ethical regulations.

## Conflicts of Interest

The authors declare no conflicts of interest.

## Data Availability

The data that support the findings of this study are available on request from the corresponding author. The data are not publicly available due to privacy or ethical restrictions.
